# Whole Genome Expression Microarray Analysis of Highly Versus Poorly Tumorigenic Murine Melanoma Cell Lines Provides Insights into Factors That Regulate Tumor Growth, Metastasis, and Immunogenicity

**DOI:** 10.3389/fimmu.2015.00452

**Published:** 2015-09-14

**Authors:** Kristian Michael Hargadon

**Affiliations:** ^1^Hargadon Laboratory, Department of Biology, Hampden-Sydney College, Hampden-Sydney, VA, USA

**Keywords:** melanoma, gene expression, microarray, pathway analysis, tumorigenicity

## Introduction

Melanoma, a cancer derived from pigment-producing melanocytes of the skin, is a disease of major public health significance. Although it is the least common form of skin cancer, melanoma is by far the most lethal due to its propensity to metastasize to a number of vital organs, including the brain, lungs, liver, and other visceral organs (1). According to the American Cancer Society’s 2015 Cancer Facts & Figures statistics, melanoma accounts for <2% of all skin cancer cases but is responsible for nearly 75% of skin cancer-related deaths, and it is predicted that melanoma will cause 9,940 deaths in the U.S. this year (2). Worldwide estimates of mortality for melanoma are that this disease is responsible for over 65,000 deaths annually (3). Moreover, recent data collected by the Surveillance, Epidemiology, and End Results (SEER) Program show that melanoma incidence rates have continued to increase over the previous 40 years (4). In the U.S. alone, the current annual costs for treatment and productivity losses associated with melanoma are near $3.3 billion (5). These numbers are even more staggering when considering a recent study by the International Agency for Research on Cancer, which ranked North America only fourth in melanoma incidence worldwide (6), thus highlighting the need to address melanoma as a global public health concern. With these statistics in mind, it is vital that we increase our understanding of the progression of melanoma so that we may improve current – and develop new – therapies for the treatment of this cancer.

The B16 murine melanoma cell line is one of the most widely used models for studying the progression and treatment of melanoma in pre-clinical settings (7, 8). Characterized by its rapid outgrowth as both primary tumors and metastatic lesions, the B16-F1 subline has also been shown to elicit dysfunctional CD8+ T cell responses (9) that mimic those frequently observed in many melanoma patients (10–12), and soluble factors derived from this tumor have been shown to alter the function of dendritic cells (DC) as well (13–15). We have recently described a chemically mutated variant of B16 melanoma, D5.1G4, that is significantly less tumorigenic and immunosuppressive than its wild-type counterpart (13, 15), though the basis for these differences between B16-F1 and D5.1G4 are poorly understood. Therefore, we sought to gain insights into factors that regulate melanoma tumorigenicity by comparing whole genome expression profiles of these murine melanomas. The microarray dataset obtained from these experiments and described herein offers a great resource for investigators wishing to study melanoma growth and progression and will likely drive the design of future experiments aimed at understanding the role of both individual gene products and entire pathways in melanoma tumorigenesis.

## Materials and Methods

### Cell lines

D5.1G4 murine melanoma cells were a generous gift of Dr. Jerry Neiderkorn (University of Texas Southwestern Medical School). B16-F1 murine melanoma cells were a generous gift of Dr. Victor Engelhard (University of Virginia). These tumor cell lines were grown in RPMI-1640 medium (Thermo Scientific, Hudson, NH, USA) supplemented with 10% fetal bovine serum (Premium Select, Atlanta Biologicals, Norcross, GA, USA), 2 mM L-glutamine, 2 grams/liter glucose, 2 grams/liter sodium bicarbonate, 100 U/ml penicillin (American Type Culture Collection, Manassas, VA, USA), and 100 μg/ml streptomycin (ATCC). All cells were grown at 37°C in 5% CO_2_ and were passaged at 80–90% confluence.

### Tumor challenge

To evaluate tumor outgrowth, 4 × 10^5^ B16-F1 or D5.1G4 melanoma cells in 0.2 ml of endotoxin-free 1× PBS were injected either subcutaneously in the nape of the neck or intravenously via the lateral tail vein. Mice injected subcutaneously were monitored every day for tumor formation. Following tumor formation, tumor area was determined every 2–3 days using digital calipers to take perpendicular diameter measurements of the tumor. Mice were euthanized once tumor area reached >300 mm^2^ or tumors became necrotic. Mice challenged intravenously were euthanized 17 days post-tumor challenge, and lungs were harvested for counting metastatic lesions under a dissecting microscope. Mice were used between 8 and 12 weeks of age, and all experiments were performed in accordance with regulatory standards and guidelines approved by the Hampden-Sydney College Animal Care and Use Committee.

### RNA isolation

Prior to RNA isolation, tumor cells (1 × 10^6^ cells/well) were plated in 6-well tissue culture plates and incubated at 37°C in 5% CO_2_. After 24 h of culture, B16-F1 and D5.1G4 melanoma cells were collected by cell scraping and centrifuged at 1,500 rpm for 5 min. Cell pellets were lysed with Buffer RLT containing β-ME, and RNA was extracted using an RNeasy Mini Kit (Qiagen, Valencia, CA, USA) according to the manufacturer’s recommendations. Following RNA extraction, samples were treated with Amplification Grade DNase I (Life Technologies, Grand Island, NY, USA) to digest any residual DNA, and RNA was quantified using an Epoch Spectrophotometer (BioTek, Winooski, VT, USA). A260/280 ratios for all samples were >2.0.

### Whole genome expression microarray

RNA samples (replicates of 3 for each melanoma cell line) were diluted to 100 ng/μl, and ~5 μg of RNA was shipped overnight on dry ice to Arraystar, Inc. (Rockville, MD, USA) for analysis using the company’s gene expression service and Agilent Mouse 4 × 44K Gene Expression Microarray v2 (Agilent Design ID 026655). RNA integrity was assessed by standard denaturing agarose gel electrophoresis. Sample labeling was performed according to the Agilent One-Color Microarray-Based Gene Expression Analysis protocol (Agilent Technology). Briefly, total RNA from each sample was linearly amplified and labeled with Cy3-UTP. The labeled cRNAs were purified using the RNeasy Mini Kit (Qiagen). The concentration and specific activity of the labeled cRNAs (pmol Cy3/μg cRNA) were measured using a NanoDrop ND-1000. One microgram of each labeled cRNA was fragmented by adding 11 μl 10× blocking agent and 2.2 μl of 25× fragmentation buffer, then heated at 60°C for 30 min, and finally 55 μl 2× GE hybridization buffer was added to dilute the labeled cRNA. One hundred microliters of hybridization solution were dispensed into the gasket slide and assembled to the gene expression microarray slide [Agilent Mouse 4 × 44K Gene Expression Microarray v2 (Agilent Design ID 026655)]. The slides were incubated for 17 h at 65°C in an Agilent hybridization oven. The hybridized arrays were washed and fixed prior to scanning. Slides were scanned on the Agilent DNA microarray scanner (G2505C) using the one-color scan setting for 1 × 44K array slides (scan area = 61 mm × 21.6 mm, scan resolution = 5 μm, dye channel set to Green, and Green PMT set to 100%).

### Data processing and analysis

Agilent Feature Extraction software (version 11.0.1.1) was used to analyze the acquired array images. Quantile normalization and subsequent data processing were performed using the GeneSpring GX v12.1 software (Agilent Technologies). After quantile normalization of the raw data, genes for which at least three out of six samples had flags in Detected (“All Targets Value”) were chosen for further data analysis. Differentially expressed genes with statistical significance were identified through Volcano Plot filtering. D5.1G4 melanoma served as the reference sample, and genes expressed at greater than or equal to twofold higher or lower levels in B16-F1 (with a *p* value ≤0.05) were defined as genes exhibiting differential expression between the two tumors with statistical significance. Pathway analysis was performed using the latest Kyoto Encyclopedia of Genes and Genomes (KEGG) database and the standard enrichment computation method.

### Data deposition

The data discussed in this publication have been deposited in NCBI’s Gene Expression Omnibus (16) and are accessible through GEO Series accession number GSE69908 (http://www.ncbi.nlm.nih.gov/geo/query/acc.cgi?acc=GSE69908). Data are freely available from this repository under file name GSE69908_RAW.tar, which contains .txt files of the normalized signal intensities for each Agilent Probe Name for all replicates of each sample.

## Results

**Figure 1:**
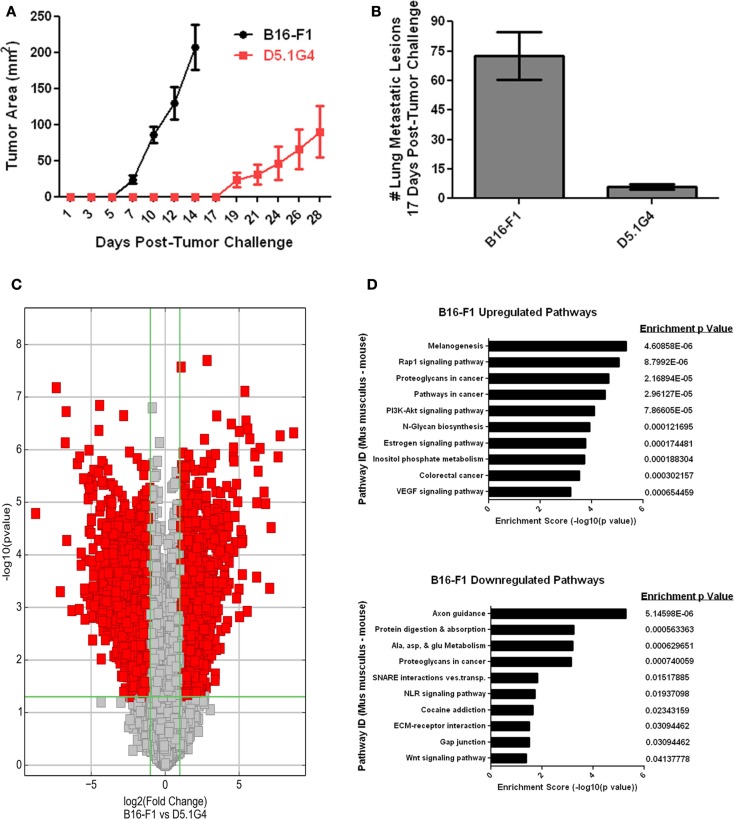
**Whole genome microarray analysis of B16-F1 and D5.1G4 murine melanomas**. C57Bl/6 mice were challenged with 4e^5^ B16-F1 or D5.1G4 melanoma cells either subcutaneously **(A)** or intravenously **(B)**. Subcutaneous tumor outgrowth was monitored over time, and lung metastatic lesions were enumerated 17 days post-tumor challenge. Data represent five mice per group and are graphed as the average with error bars designating standard error of the mean. Data shown are representative of three independent experiments. **(C)** RNA isolated from each respective melanoma cell line was used for whole genome microarray expression analysis as described in Materials and Methods. Volcano plot filtering was performed to identify genes that were differentially expressed with statistical significance between the B16-F1 and D5.1G4 melanomas. **(D)** KEGG pathway analysis was performed to identify biological pathways significantly enriched with differentially upregulated and downregulated genes in B16-F1.

B16-F1 is a well-characterized murine melanoma cell line that models the growth, metastasis, and disruption of anti-tumor immunity often exhibited by aggressive melanomas in many patients with this disease. D5.1G4 is a chemically mutated variant of B16 that is significantly less tumorigenic than its wild-type counterpart, both with respect to its growth as primary tumors in the skin (Figure 1A) and with regard to its growth as metastatic lesions in the lungs (Figure 1B). In order to gain insight into factors that regulate melanoma growth and progression, RNA was isolated from these two melanoma cell lines for whole genome expression microarray analysis with the Agilent Mouse 4 × 44K Gene Expression Microarray v2 (Agilent Design ID 026655) that profiles the expression of 39,430 genes. Volcano Plot filtering was used to identify differentially expressed genes exhibiting greater than or equal to twofold upregulation or downregulation in B16-F1 melanoma as compared to D5.1G4 melanoma (Figure 1C). The dataset obtained from this analysis, accessible through the NCBI GEO data repository (16) at http://www.ncbi.nlm.nih.gov/geo/query/acc.cgi?acc=GSE69908, revealed statistically significant upregulation of 1,462 genes and statistically significant downregulation of 1,935 genes in B16-F1 melanoma. In order to better understand how these differences in gene expression might impact the biological properties of these melanoma cell lines, KEGG Pathway analysis was performed to identify biological pathways in which there was a significant enrichment of differentially expressed genes. In all, 71 KEGG pathways exhibited a statistically significant enrichment (*p* < 0.05) in upregulated genes in B16-F1, and 15 KEGG pathways exhibited a statistically significant enrichment in downregulated genes in this melanoma. The pathways with the 10 most significant enrichments scores for both upregulated and downregulated genes in B16-F1 are shown in Figure 1D, and the pathway-associated genes exhibiting a greater than or equal to twofold change in expression in this tumor versus the D5.1G4 melanoma are listed in Table 1.

**Table 1:** **Differentially expressed genes associated with pathways with highest enrichment scores**.

Pathway ID* (*Mus musculus*)	Differentially expressed genes associated with indicated KEGG pathway
**B16-F1 upregulated pathways**
Melanogenesis	*Adcy7, Adcy9, Calm1, Creb3l1, Dvl2, Fzd2, Fzd6, Fzd7, Fzd9, Kit, Map2k1, Mapk1, Mc1r, Nras, Tcf7, Tcf7l1, Tyr, Wnt3a, Wnt9b*
Rap1 signaling pathway	*Adcy7, Adcy9, Adora2b, Akt2, Angpt2, Arap2, Calm1, Efna1, Efna4, Fgfr1, Itgb1, Kit, Lpar2, Map2k1, Mapk1, Mapk11, Nras, P2ry1, Pard6a, Pfn4, Pik3cd, Plcg1, Rac1, Rapgef3, Rasgrp2, Rasgrp3, Rgs14, Rhoa, Sipa1l1, Thbs1*
Proteoglycans in cancer	*Akt2, Col1a1, Ctsl, Ddx5, Eif4b, Fgfr1, Fzd2, Fzd6, Fzd7, Fzd9, Grb2, Hpse, Itgav, Itgb1, Kit, Lpar2, Map2k1, Mapk1, Mapk11, Nras, Nudt16l1, Pik3cd, Plcg1, Plcg2, Rac1, Rhoa, Tgfb1, Thbs1, Tlr2, Wnt3a, Wnt9b*
Pathways in cancer	*Akt2, Axin1, Cdk2, Col4a1, Col4a4, Dvl2, Egln1, Epas1, Fgfr1, Fos, Fzd2, Fzd6, Fzd7, Fzd9, Grb2, Itgav, Itgb1, Kit, Lama5, Lamb1, Map2k1, Mapk1, Msh3, Nras, Pik3cd, Plcg1, Plcg2, Pten, Rac1, Rhoa, Rxrg, Tcf7, Tcf7l1, Tgfb1, Traf4, Wnt3a, Wnt9b, Xiap*
PI3K–Akt signaling pathway	*Akt2, Angpt2, Atf2, Cdk2, Col1a1, Col4a1, Col4a4, Creb3l1, Efna1, Efna4, Eif4b, Fgfr1, Gnb4, Grb2, Itga1, Itga7, Itgav, Itgb1, Kit, Lama5, Lamb1, Lpar2, Map2k1, Mapk1, Nr4a1, Nras, Pck2, Pik3cd, Ppp2r1a, Ppp2r2b, Ppp2r3d, Prakaa1, Pten, Rac1, Thbs1, Them4, Tlr2, Ywhaz*
N-glycan biosynthesis	*Alg14, Alg5, Alg8, B4galt2, B4galt3, Fut8, Man1c1, Man2a1, Rft1, St6gal1, Stt3a*
Estrogen signaling pathway	*Adcy7, Adcy9, Akt2, Atf2, Calm1, Creb3l1, Fos, Grb2, Hspa8, Itpr3, Map2k1, Mapk1, Nras, Pik3cd, Prkcd, Shc4*
Inositol phosphate metabolism	*Aldh6a1, Inpp5b, Ippk, Isyna1, Minpp1, Pi4k2b, Pik3cd, Pikfyve, Pip4k2b, Plcg1, Plcg2, Pten*
Colorectal cancer	*Akt2, Axin1, Fos, Map2k1, Mapk1, Msh3, Pik3cd, Rac1, Rhoa, Tcf7, Tcf7l1, Tgfb1*
VEGF signaling pathway	*Akt2, Map2k1, Mapk1, Mapk11, Nras, Pik3cd, Plcg1, Plcg2, Ppp3cc, Rac1, Sphk1*
**B16-F1 downregulated pathways**
Axon guidance	*Ablim1, Dcc, Epha1, Epha2, Epha3, Epha4, Epha7, Ephb6, Gnai1, Plxna4, Plxnb3, Ptk2, Rgs3, Robo1, Sema3c, Sema3f, Sema4d, Sema4f, Sema6a, Sema6c, Slit2, Srgap1*
Protein digestion and absorption	*Coll11a2, Col17a1, Col18a1, Col1a2, Col2a1, Col4a2, Col4a4, Col9a1, Mme, Prcp, Slc7a9, Try4, Try5*
Alanine, aspartate, and glutamate metabolism	*Abat, Ass1, Ddo, Gfpt1, Gfpt2, Gls, Glul, Il4i1*
Proteoglycans in cancer	*Ank3, Araf, Camk2b, Camk2d, Cav2, Cd44, Col1a2, Fas, Fgf21, Fgf9, Fn1, Fzd8, Gpc1, Hbegf, Hspg2, Igf1r, Mapk13, Mras, Mtor, Ppp1r12a, Ptk2, Sdc4, Slc9a1, Wnt10a, Wnt5b, Wnt6*
SNARE interactions in vesicular transport	*Stx11, Stx16, Stx17, Stx3, Vamp1, Vamp5*
NOD-like receptor signaling	*Ccl2, Ccl5, Map3k7, Mapk13, Naip7, Nlrp1a, Nod1, Tnfaip3*
Cocaine addiction	*Cdk5r1, Creb3, Gnai1, Gnas, Grm2, Maoa, Slc18a1*
ECM-receptor interaction	*Cd44, Col11a2, Col1a2, Col2a1, Col4a2, Col4a4, Fn1, Gp9, Hspg2, Sdc4*
Gap junction	*Gja1, Gnai1, Gnas, Gucy1a3, Gucy1b2, Pdgfrb, Plcb4, Tuba1a, Tubb2b, Tubb3*
Wnt signaling pathway	*Apc, Camk2b, Camk2d, Fzd8, Map3k7, Nfatc1, Nkd1, Nkd2, Plcb4, Porcn, Vangl1, Wnt10a, Wnt5b, Wnt6*

*Pathways with the 10 highest enrichment scores for differentially upregulated and downregulated genes in B16-F1 as compared to D5.1G4. Genes listed are those within the indicated pathways that exhibit a greater than or equal to twofold change in expression in B16-F1 with a *p* value <0.05.

## Discussion

The gene expression microarray data described herein and available for further inspection and analysis at the publicly accessible NCBI GEO repository are a valuable tool for investigators wishing to study the roles played by individual genes and biological pathways in regulating melanoma tumorigenicity. These data are likely to be useful for those wishing to gain insights into melanoma-associated genes that influence various facets of tumor growth and progression, including cell cycle regulation, survival, cell adhesion and motility, tissue invasion and metastasis, and immune evasion. Our laboratory has been particularly interested in this latter phenomenon, as CD8+ T cell responses to well-established B16 melanoma have been shown to exhibit the type of dysfunction often associated with anti-tumor T cell responses in melanoma patients (9–12). Of note, our microarray data revealed a 16.77-fold upregulation (*p* < 0.0001) of *Tgfb1* gene expression in B16-F1 as compared to its poorly tumorigenic D5.1G4 counterpart. It is therefore possible that melanoma-derived TGFβ1 suppresses CD8+ T cell effector function, leading to tumor immune escape and enhanced outgrowth. We have also recently shown that B16-F1-derived TGFβ1 alters that maturation and activation of tissue-resident DC and promotes their acquisition of a phenotype that is likely to favor tumor progression (15). Tumor-associated glycans have also been reported to promote immunosuppressive activity in DC (17), and KEGG pathways relating to glycan biosynthesis and cancer-associated proteoglycans were enriched with several genes exhibiting significant upregulation in B16-F1 (Table 1). It is interesting to speculate that overactivation of these and related immunoregulatory genes in B16-F1 may suppress cytotoxic T cell function or preclude efficient stimulation of CD8+ T cells by tumor-associated DC, thereby promoting tumor immune escape and enhanced outgrowth.

In addition to the role that overexpressed genes might play in promoting immune escape by B16-F1 melanoma, it is likely that downregulation of certain genes by B16-F1 may also contribute to escape from anti-tumor immune responses, even those that are robustly activated and exhibit significant effector function. For instance, our microarray data revealed significant downregulation of *Cd72* (9.93-fold, *p* < 0.001) and *Fas* (9.91-fold, *p* < 0.01) gene expression in B16-F1 melanoma. CD72 functions as a cell adhesion and costimulatory ligand for the CD5 receptor on T cells (18), and its downregulation may limit high-affinity interactions between melanoma cells and tumor antigen-specific T cells. Likewise, melanoma-associated downregulation of Fas may promote tumor escape from activated tumor-specific T cells expressing FasL. Such findings might explain B16-F1 melanoma outgrowth in the face of the functional anti-tumor CD8+ T cell responses that are elicited against this tumor in early stages of its growth (9), and subsequent tumor progression could then be compounded by the overexpression of genes with active immunosuppressive functions as described above. It is also interesting that there is a significant enrichment of downregulated genes associated with the NOD-like receptor signaling pathway in B16-F1 melanoma. Downregulation of the *Ccl2* and *Ccl5* genes associated with this pathway may preclude efficient recruitment of various immune effectors into tumors or tumor-bearing tissue. Downregulation of other genes associated with this pathway, such as the *Nod1* gene that promotes inflammation and *Nlrp1* gene that functions to induce apoptosis, would also be expected to contribute to immune escape, enhanced survival, and the overall protumorigenic nature of this melanoma.

In conclusion, this comparative analysis of the whole genome expression profiles for the highly tumorigenic B16-F1 and poorly tumorigenic D5.1G4 melanomas offers a resource of data that provides significant insights into factors that regulate melanoma tumorigenicity. Those genes and pathways highlighted in this report are just a sample of the many that are likely to be of interest to tumor immunologists and cancer biologists, and it is hoped that the data obtained from this study will drive future investigation into the dysregulation of individual genes and biological pathways in melanoma that will enhance our understanding of this tumor’s progression and inform the design of new and improved therapies for the treatment of this cancer.

## Author Contributions

KH was responsible for all aspects of this article and the work related to it.

## Conflict of Interest Statement

The author declares that the research was conducted in the absence of any commercial or financial relationships that could be construed as a potential conflict of interest.

## Funding

This research was supported by funding from Virginia’s Commonwealth Health Research Board (Grant #375-01-14) and a Virginia Academy of Science Jeffress Research Grant from the Thomas F. Jeffress and Kate Miller Jeffress Memorial Trust (Grant #J-1018).

## References

[1] Leung AM Hari DM Morton DL. Surgery for distant melanoma metastasis. Cancer J (2012) 18:176–84. 10.1097/PPO.0b013e31824bc981 22453019 PMC3346255

[2] American Cancer Society. Cancer Facts & Figures 2015 (2015). Available from: http://www.cancer.org/acs/groups/content/@editorial/documents/document/acspc-044552.pdf. Accessed June 30, 2015.

[3] Lucas R McMichael T Smith W Armstrong B Solar Ultraviolet Radiation: Global Burden of Disease from Solar Ultraviolet Radiation. Environmental Burden of Disease Series, No. 13. Geneva: World Health Organization (2006).

[4] Howlader N Noone AM Krapcho M Garshell J Miller D Altekruse SF, editors. SEER Cancer Statistics Review, 1975-2012. Bethesda, MD: National Cancer Institute (2015) [based on November 2014 SEER data submission, posted to the SEER web site, April 2015]. Available from: http://seer.cancer.gov/csr/1975_2012/

[5] Guy GP Machlin SR Ekwueme DU Yabroff KR. Prevalence and costs of skin cancer treatment in the U.S., 2002-2006 and 2007-2011. Am J Prev Med (2015) 48:183–7. 10.1016/j.amepre.2014.08.036 25442229 PMC4603424

[6] Riker AI Zea N Trinh T. The epidemiology, prevention, and detection of melanoma. Ochsner J (2010) 10:56–65. 21603359 PMC3096196

[7] Overwijk WW Restifo NP B16 as a mouse model for human melanoma. In: Coligan JE, editors. Current Protocols in Immunology (2001). Chapter 20: Unit 20.1. 10.1002/0471142735.im2001s39 PMC2763508 18432774

[8] Ya Z Hailemichael Y Overwijk W Restifo NP Mouse model for pre-clinical study of human cancer immunotherapy. In: Coligan JE, editors. Current Protocols in Immunology, Vol. 108 (2015). 20.1.1–43. 10.1002/0471142735.im2001s108 25640991 PMC4361407

[9] Hargadon KM Brinkman CC Sheasley-O’Neill SL Nichols LA Bullock TNJ Engelhard VH. Incomplete differentiation of antigen-specific CD8 T cells in tumor-draining lymph nodes. J Immunol (2006) 177:6081–90. 10.4049/jimmunol.177.9.6081 17056534

[10] Anichini A Scarito A Molla A Parmiani G Mortarini R. Differentiation of CD8+ T cells from tumor-invaded and tumor-free lymph nodes of melanoma patients: role of common gamma-chain cytokines. J Immunol (2003) 171:2134–41. 10.4049/jimmunol.171.4.2134 12902520

[11] Mortarini R Piris A Maurichi A Molla A Bersani I Bono A Lack of terminally differentiated tumor-specific CD8+ T cells at tumor site in spite of antitumor immunity to self-antigens in human metastatic melanoma. Cancer Res (2003) 63:2535–45. 12750277

[12] Zippelius A Batard P Rubio-godoy V Bioley G Lie D Lejeune F Effector function of human tumor-specific CD8 T cells in melanoma lesions: a state of local functional tolerance. Cancer Res (2004) 64:2865–73. 10.1158/0008-5472.CAN-03-3066 15087405

[13] Hargadon KM Forrest OA Reddy PR. Suppression of the maturation and activation of the dendritic cell line DC2.4 by melanoma- derived factors. Cell Immunol (2012) 272:275–82. 10.1016/j.cellimm.2011.10.003 22051048

[14] Hargadon KM Ararso YT Forrest OA Harte CM Melanoma-associated suppression of the dendritic cell lines DC2.4 and JAWSII. Am J Immunol (2012) 8:179–90. 10.3844/ajisp.2012.179.190

[15] Hargadon KM Bishop JD Brandt JP Hand ZC Ararso YT Forrest OA. Melanoma-derived factors alter the maturation and activation of differentiated tissue-resident dendritic cells. Immunol Cell Biol (2015). 10.1038/icb.2015.58 26010746

[16] Edgar R Domrachev M Lash AE. Gene expression omnibus: NCBI gene expression and hybridization array data repository. Nucleic Acids Res (2002) 30:207–10. 10.1093/nar/30.1.207 11752295 PMC99122

[17] Nonaka M Ma BY Murai R Nakamura N Baba M Kawasaki N Glycosylation-dependent interactions of C-type lectin DC-SIGN with colorectal tumor-associated lewis glycans impair the function and differentiation of monocyte-derived dendritic cells. J Immunol (2008) 180:3347–56. 10.4049/jimmunol.180.5.3347 18292560

[18] Van de Velde H von Hoegen I Luo W Parnes JR Thielemans K. The B-cell surface protein CD72/Lyb-2 is the ligand for CD5. Nature (1991) 351:662–5. 10.1038/351662a0 1711157

